# From Which Direction Does the Empire Strike (Back)?

**DOI:** 10.3389/fpsyg.2021.625554

**Published:** 2021-04-29

**Authors:** Katharina Theresa Halicki, Moritz Ingendahl, Maren Mayer, Melvin John, Marcel Raphael Schreiner, Michaela Wänke

**Affiliations:** Department of Consumer and Economic Psychology, School of Social Sciences, University of Mannheim, Mannheim, Germany

**Keywords:** spatial agency bias, mental representation, attack, defense, spatial orientation

## Abstract

In cultures with left-right-script, agentic behavior is mentally represented as following a left-to-right trajectory, an effect referred to as the Spatial Agency Bias (SAB, Suitner and Maass, 2016). In this research, we investigated whether spatial representations of activities are universal across activities by analyzing the opposite concepts of “attack” and “defense”. Both behaviors involve similar actions (e.g., fighting) but may differ in perceived agency. Moreover “defense” is necessarily always a response to an attack and may therefore be represented by a trajectory in the opposite direction. Two studies found the classic SAB for activities representing attacking but a reduction (Study 1) and reversal (Study 2) for activities involving defense. Although the spatial representation of defense on the right was much weaker and less unequivocal than that of attack on the left, the results suggest that the spatial representations of defense and attack are located in different positions. Apparently not all actors and all activities are spatially represented on the left with a left-to-right trajectory but position and direction depend on the perceived agency. Directions for future research and applications of our findings are discussed.

## Introduction

Mental representations often involve a spatial dimension (for reviews see Smith and Semin, 2004). For example, people associate power and high status with an upper vertical location (Giessner and Schubert, 2007) and political orientations are mapped on a horizontal axis from “left” to “right” (van Elk et al., 2010). Likewise, numbers and magnitudes are ordered along a horizontal axis (Spatial-Numerical Association of Response Codes, SNARC effect, Dehaene et al., 1993). Central to the present paper, also the mental representation of actions or people being involved in actions is spatially biased. This so-called Spatial Agency Bias (SAB) describes that in cultures with left-to-right script direction the agent, the person acting, is more likely to be represented on the left and the person receiving the action on the right (for an overview see Suitner and Maass, 2016)^1^. In the present studies, we investigate whether all actions or only specific types of actions are associated with this left-to-right bias or if some behaviors even underlie a reversed spatial bias. Before we turn to possible moderations we will first give an overview of the SAB.

According to the SAB, the predominant writing system employed in a given cultural context influences how humans envisage actions (Suitner and Maass, 2016). Two aspects, namely a linguistic component as well as script direction influence how we mentally represent agentic behavior.

The linguistic component exerts its influence through the subject-object order. In many languages, such as most Indo-European languages like German, English, or Italian, the grammatical subject syntactically precedes the grammatical object (Bettinsoli et al., 2015). In standard active sentences, this means that the thematic agent is mentioned before the person receiving the action (e.g., “Tom kicks George”).

Moreover, the visual motor component of reading and writing internalizes the script direction. Reading and writing from left to right in combination with the thematic agent being written left to the recipient causes an association of agency with the left side and therefore also an association of agency and a rightward orientation. Any stimulation of the motor system would then promote the use of a spatial bias in which people construe the movements and actions accordingly (Suitner and Maass, 2016). This results in an overgeneralized bias that actions are mentally represented spatially biased with a rightward moving trajectory. Notably, for people socialized in cultures with the reverse script direction the bias reverses (Maass and Russo, 2003; Dobel et al., 2007; Maass et al., 2007, 2014). For instance, Italian participants (reading from left to right) tend to draw an agent on the left-hand side of a picture and Iraqi participants (reading from right to left) on the right-hand side (Maass et al., 2014).)

Relying on the name of the bias, one should expect that it is not activities as such, but the concept of agency that is associated with the rightward (left-to-right) bias. The authors of the SAB refer to agency as its “essential features, namely, *acting or having the capacity to act autonomously in a given environment*” (Suitner and Maass, 2016, p. 248). While this definition leaves room for a wide variety of actions and behaviors that could potentially be affected by the bias one may also wonder whether actions undertaken in response to others’ actions are fully autonomous. Possibly, the fact that they are not completely originating from the actor may weaken the bias. One should expect that such actions that only occur in response to another action (e.g., answering a question, accepting or rejecting a gift) are even associated with an opposing bias, namely following a right-to-left trajectory.

Moreover, as stated by Hitlin and Elder (2007, p. 171): “the concept of “agency” is certainly influenced by Western conceptions of the actor”. Other definitions of agency involve instrumentality, ambition, dominance, competence, efficiency and control over the action (Bandura, 1989; Abele and Wojciszke, 2007; Wojciszke et al., 2009). This means that some behaviors or actions might be more closely related to agency than others and might therefore be more strongly affected by the bias. In this regard, one may wonder to what extent these other aspects of agency moderate the spatial representations of actions.

To investigate whether the spatial representations of activities are universal across activities we chose the opposite concepts of “attack” and “defense”. Both involve similar actions (e.g., fighting) but may differ in perceived agency. When we conceive agency in terms of dominance over others, ambition, individualism, masculinity, self-interest and efficiency (Bandura, 1989; Abele and Wojciszke, 2007; Wojciszke et al., 2009), attacking clearly meets the criteria of high agency and could be a prime example for agentic behavior. Therefore, we expect that attacking is mentally represented according to the script direction, i.e., from left to right in languages writing from left to right (e.g., German, English, and Italian, etc.) but from right to left in languages writing from right to left.^2^

Indeed, previous research provides preliminary evidence for this assumption. In one study, either Italian or Iraqi participants were asked to draw two scenes of interactions between two people. One scene should illustrate aggressive behavior. Italian participants drew the agent more often on the left-hand side in a picture. Iraqi participants, cohering to their habitual script direction from right to left, drew the agent more often on the right-hand side (Maass et al., 2014). Similar effects could also be obtained in a picture-matching task. Among four scenes, one included the description that “Claudio kicks Gianni”. In these studies, participants with a rightward script more often chose the picture with the person kicking on the left-hand side and participants with a leftward script preferred the picture with the person kicking being presented on the right-hand side (Maass et al., 2014).

Besides preferences for position and moving trajectory, studies also indicate that participants draw inferences based on position and direction of movement. For example, Maass et al. (2007) also show that within a culture with left-to-right script direction violent movie scenes are perceived as more violent if the act of violence is presented with a left-to-right trajectory. Even though it is not explicitly stated in these studies who is attacking and who is defending, aggression itself–being related to attack–seems to be associated with a spatial orientation from left to right and appears to be more agentic, when shown in this position or trajectory.

When it comes to defending, the predictions are less clear. On the one hand, there are reasons for a placement on the left (in cultures with a left-to-right script direction). First, defending involves an agent who engages in an activity, which would suggest a placement on the left. Second, defending involves similar activities as attacking, for example fighting, combatting and battling. Moreover, in fiction (e.g., 300 or Braveheart) defending oneself or victims of an attack or an oppressive system often seems to be portrayed in a quite heroic and agentic manner. Hence, it could also be associated with ambition, self-interest and efficiency and could be seen as agentic. This would predict a similar left-to-right bias as for attacking.

On the other hand, an important part of agency or the role of the agent is control over the action (Bandura, 1989; Hitlin and Elder, 2007; Suitner and Maass, 2016). A defensive action, however, is characterized by reaction and therefore, it is less likely to be seen as self-determined or having control over the event. Further, defending could be seen as the attempt of keeping the *status quo* or just holding the position, which resembles aspects of low agency. Thus, one would expect a reduced left-to-right bias compared to attacking. Additionally, when attacks are expected to follow a left-to-right trajectory, one should expect that a defensive action (being the reaction to an attack) should consequently be represented in the opposite direction and therefore reasonably originate from the right-hand side.

We tested a possible moderation of the SAB for the two different activities attacking and defending in two studies using two paradigms prominent in the SAB research. In Study 1 we had participants create pictures illustrating situations verbally described as attack or defense. Picture drawing (or selecting pictures) to illustrate a described action has been widely used in this domain (Maass et al., 2014; Suitner et al., 2017). However, complicating things, recent research showed that the left position is also the place for the most relevant protagonist of the verbal description independent of whether this is the agent or not (Halicki et al., 2021). Most often this is the grammatical subject. According to this relevance bias in a scene illustrating attack, the attacker should be placed left, and in a scene illustrating defense the defender should be placed on the left. The same applies if the spatial representation is determined by who is performing the action. Again, based on the relevance bias or if the SAB is determined by who is performing the action, we would expect no difference for a defender or attacker. Only if we assume that the spatial representation is determined by the extent of perceived agency in terms of autonomy, dominance or power one would predict differences between the placement of attacker and defender, with the left bias more strongly pronounced for the attacker than for the defender. Still, in this paradigm, we cannot rule out a potential effect of the relevance bias possibly weakening the effects of differences in agency.

Study 2 was meant to isolate the concepts of attack and defense more purely. Therefore, we adapted a paradigm developed by Suitner et al. (2017) and asked participants to place synonyms of the two concepts on arrows following a left-to-right or right-to-left direction.

## Study 1

In a first approach to investigate how participants mentally represent the spatial orientation of attacking and defending, we implemented a new online art-paradigm where participants had to allocate the position of an attacking and defending party.

### Materials and Methods

#### Participants

We conducted an *a priori* power analysis for a binomial test as a rough and conservative approximation for how many participants were necessary. For *p*_*H*0_ = 0.5 and *p*_*H*1_ = 0.65, α = 0.05, the desired power of 80% was achieved with *N* = 67. Due to uncertainty regarding the actual effect sizes, we decided to collect at least 90 participants.^3^ Participants were recruited via social networks (e.g., Facebook groups), mailing lists and the participation website from a German Pop Psychology magazine called “Psychologie Heute”. As a reward for participating, participants could choose between a lottery to win one of two Amazon vouchers worth 10€ or a donation of 20cts to the German aid organization “Brot für die Welt” by the authors. In total, 115 participants finished the questionnaire. We excluded 15 participants who stated being familiar with the SAB or spoke a language with a right-to-left script direction. Thus, our final sample consisted of 100 participants (63% female, 35% male, 2% other or not specified, *M*_*age*_ = 25.76, *SD*_*age*_ = 7.19). They were mostly students (69%) and native Germans (92%).

#### Design and Procedure

Participants created eight pieces of art depicting scenes from galactic wars involving battle space ships. We varied whether attack or defense should be depicted. Because it is well likely that what is placed first is placed on the left (following script direction) we pre-selected for each scene with which spaceship participants had to begin, counterbalancing between the attacking and the defending space ship. This resulted in a 2 (scene: attack vs. defense) × 2 (preselection: attacking space ship preselected vs. defending space ship preselected) experimental within-participants design. Every participant completed two trials of each condition, resulting in a total of eight measurements per participant (four attack trials and four defense trials).

Participants were told that they should slip into the role of being “space artists” in a distant future and that they were preparing an exhibition of art depicting intergalactic conflict for an intergalactic art gallery. On the following page, participants could familiarize themselves with the design tool to create the art. Afterward they went on to create their eight pieces of art.

For each piece, the context information was provided in the following way:

*Since the [year] age there has been a war between the [galaxy A] and the [galaxy B] in [location]. The next picture should depict a scene from this conflict and will be titled: The [attack vs. defense word] of the [galaxy A] in the [year] age, [location]. Think about how you want to design the picture. Once you made some thoughts on the design, please click on “next” to start designing!*

**FIGURE A1:**
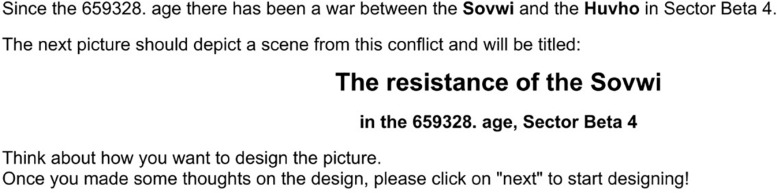
Exemplary context information of a scene in Study 1.

For the year and the location, an element from a randomized set of eight items was chosen (e.g., year: 659328. age; location: Sector Beta 4). Since participants were confronted with eight trials in total, the names of galaxies were chosen from a randomized set of 16 non-words (e.g., HUVHO and SOVWI) so that galaxy names appeared only once per participant (e.g., a participant received: “war between the HUVHO and the SOVWI […]. The resistance of the SOVWI […]” and next “war between the RINCE and the TESHE […]. The attack of the RINCE […]”). For an example scene see Appendix Figure A1. For the scene descriptions we used four attack and four defense words that can be found in Appendix Table A1. To ensure that participants read the instructions in each trial these were displayed for 5 s before the “next” button appeared and participants could begin (of course they could also spend more time reading the instructions). This was meant to give participants time to construe a mental image of the scene before they had to create it.

**FIGURE A2:**
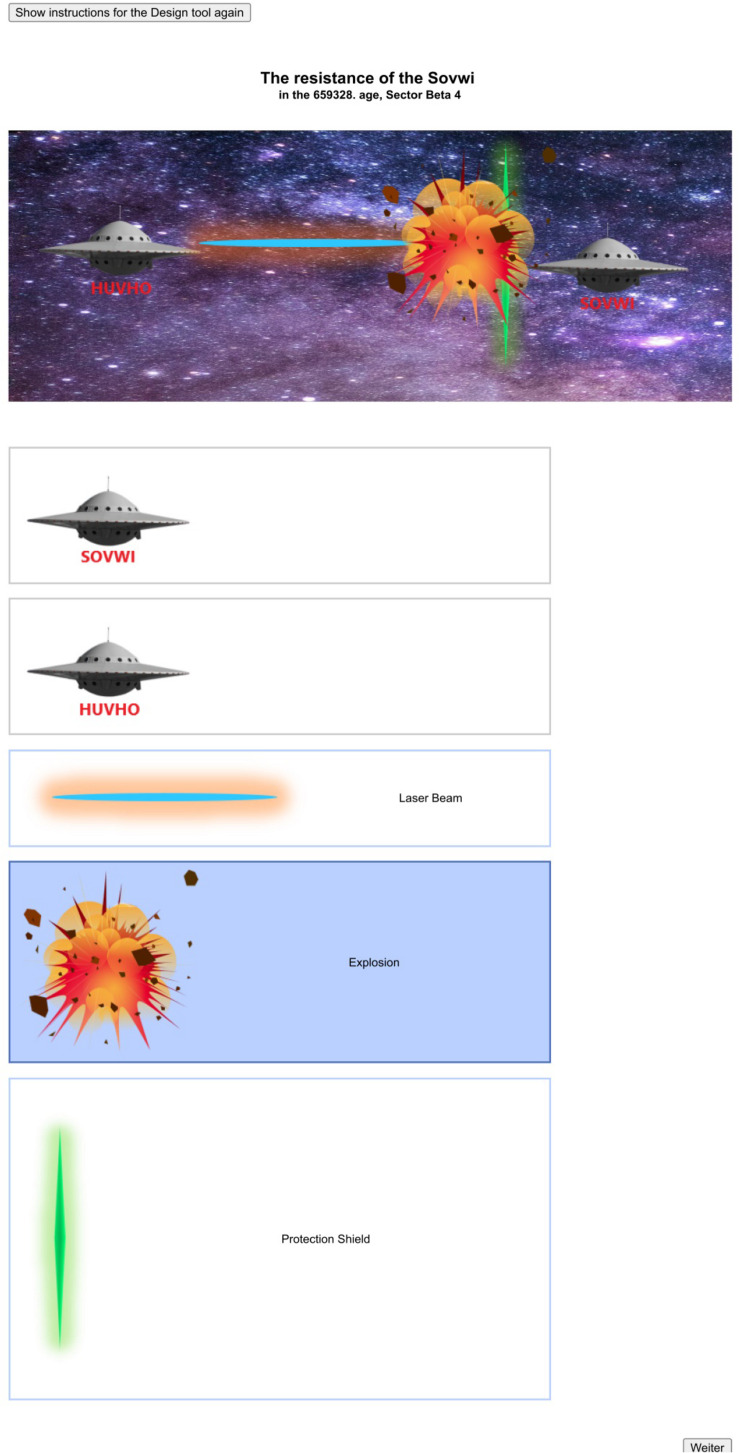
Exemplary result of a galactic artwork in Study 2.

Proceeding to the actual task, a space image with the dimensions 800 × 300 pixels was presented as a background. The title, describing the scene as attack or defense, appeared again above the space image. Underneath the background image, participants could see all the objects available. They had to click on an icon to place it on the background. There were always two spaceships, one for each of the conflicting galaxies, with the name of the respective galaxy printed underneath the space ship icon. Participants could not choose freely with which icon they wanted to start but the first icon was preselected (counterbalanced as explained above). Additional items such as laser beams, protection shields and explosions could be placed in unlimited number. Participants could easily rearrange or remove icons once placed on the background image. For an example scene see Appendix Figure A2.

We assessed the coordinates of the placed items to code which space ship icon was positioned on the left-hand side and which on the right-hand side. Once the minimum requirements for the artwork were fulfilled (positioning both space ships), participants could see the “next” button and were allowed to proceed to the next artwork. All graphics in this study were retrieved from the online image database pixabay (CC0 Creative Commons license).

After designing all eight galactic images, we assessed sociodemographic data and asked participants if they had any suspicions about the purpose of the study or if there were any reasons against using their data.

### Results

As the positioning of the eight scenes was nested within participants, we conducted a binary logistic regression analysis by means of Generalized Estimating Equations (GEE, Wilson and Lorenz, 2015). The coding of the position of the target icon (attacking space ship in the attack scene and defending space ship in the defense condition) served as our criterion variable (target space ship on the right = 0; target space ship on the left = 1). We dummy coded the scene type (attack = 0; defense = 1) and effect coded preselection (other space ship preselected = −1; target space ship preselected = 1) which were included as predictors, as well as their interaction term.

In line with our hypothesis, in the attack condition participants predominantly positioned the target (in this case the attacking space ship) left to the other space ship [66.3%, *b* = 0.69, *SE* = 0.13, Wald’s-χ^2^(1) = 26.37, *p* < 0.001, OR = 1.99, 95% CI (1.53, 2.60)].

In the defense condition, participants positioned the target icon (in this case the defending space ship) less likely left to the other space ship than in the attack condition [51.5%, *b* = −0.63, *SE* = 0.18, Wald’s-χ^2^(1) = 12.03, *p* = 0.001, OR = 0.53, 95% CI (0.37; 0.76)]. A simple intercept analysis revealed that there was no positional bias in the defense condition [*b* = 0.06, *SE* = 0.13, Wald’s-χ^2^(1) = 0.24, *p* = 0.626, OR = 1.07, 95% CI (0.83; 1.38)], neither for the left nor the right side.

Further, we found a significant effect for placement order, indicating that, as we had anticipated, the ship that had to be placed first was more likely to be placed on the left than the second ship [*b* = −0.63, *SE* = 0.18, Wald’s-χ^2^(1) = 12.03, *p* = 0.001, OR = 0.53, 95% CI (0.37; 0.76)]. This, however, was independent whether the defending or attacking ship had to be placed first, [no interaction between scene type and pre-selection: *b* = 0.14, *SE* = 0.13, Wald’s-χ^2^(1) = 1.19, *p* = 0.275, OR = 1.15, 95% CI (0.89; 1.48)]. When the defending ship had to be placed first it was less likely to be placed on the left side compared to the condition where the attacking ship had to be placed first (63 vs. 73.5%), *z* = −2.29, *p* = 0.022. Also when the defending ship had to be placed second it was less likely to be placed on the left side compared to the condition where the attacking ship had to be placed second (40 vs. 59%), *z* = −3.78, *p* < 0.001.

### Discussion

As predicted, we find clear support for the spatial bias for attack, thus replicating SAB findings with a specific agentic concept. We also find that despite similar concepts involved in attacking and defending, the SAB vanished when the scene was depicting a battle of defense. Apparently defense is spatially represented differently than attack. This is remarkable insofar as a mere relevance bias (Halicki et al., 2021) would not have predicted any differences between the attack and defense scenes. According to the relevance bias, the defending ship in the defense condition and the attacking space ship in attack scenes should have been placed on the left. The difference also speaks against the notion that it is merely performing an activity that causes the acting protagonist to be placed on the left. Rather it seems to lie in the nature of the specific activities defending and attacking that caused a different spatial representation. Presumably, defending is less associated with agency dimensions (e.g., autonomy, dominance, and control) than attacking and this translates into a less pronounced left bias.

Despite providing initial evidence for our reasoning, there are also several issues with the paradigm used in Study 1: Differences in placement may also have been fostered by placing both attacker and defender into one picture. It may well be the case that the defender is only more likely to be placed on the right if the left is reserved for the more agentic attacker. While this is perfectly in line with the notion that differences in the extent of agency produce differences in the spatial representation it is unclear whether the differences would have emerged in isolation as well. Note, however, that imagining defense almost inevitably conjures the image of an attacker and the construct of defense prerequires an existing or expected attack. In this respect one could expect even stronger effects with the defending ship being placed more clearly on the right. However, we did not find a reversal but merely an about 50:50 placement of defense on the right and on the left. Possibly this reflects that defense is more ambiguous. Some people may interpret it as belligerent and battling conforming to higher agency and others as shielding, protecting and resisting involving less agency. Alternatively, the relevance bias may possibly have diluted a reversal with its opposite influence.

Study 2 was designed to isolate the two concepts, attack and defense, from any context that could trigger additional influences.

## Study 2

In Study 2 we adapted a method established by Suitner et al. (2017) for SAB research. Participants were presented with individual words associated with the two concepts of attack and defense and were asked to allocate these words on arrows directed from left-to-right or right-to-left. This more abstract task avoids having to place attacker and defender in the same picture which might have boosted the result of Study 1. It also circumvents a possible relevance bias (Halicki et al., 2021), which may have hampered a placement on the right. This context-free paradigm allowed us to get more closely to the mental trajectory of the defense and attack actions.

### Materials and Methods

#### Participants

We used the same power analysis as in Study 1 for determining sample size^3^. Participants were recruited via social networks (e.g., Facebook groups) and a German online community called surveycircle which is a website to find study participants^4^. In total, 117 participants finished the questionnaire. We excluded 10 participants who did not process the study on a PC, Laptop or Tablet (smaller displays could not guarantee a correct graphical representation), two participants that were not fluent in German and one participant that only created missing data. This resulted in a final sample of *N* = 104 participants (70.2% female, 26% male, 3.8% other or not specified, *M*_*age*_ = 30.38, *SD*_*age*_ = 13.85).

#### Design and Procedure

We varied the theme (attack word vs. defense word) within participants. In addition, we also tested the spatial positioning for the verbs “agieren” (to act/to operate) and “reagieren” (to react) to test the assumption of a left-to-right bias for words describing an act in response to a prior act.

We ran the study completely in an online format (using the platform soscisurvey).^5^ Participants were informed that research assumes that some words are associated with a spatial orientation and that we aimed to test this assumption for a set of words. Below, participants were presented with a list of 34 words and were told that their task would be to decide whether they think each word fits more with a left-to-right arrow or a right-to-left arrow.

Sixteen of the words were associated with attacking and sixteen words were associated with defending. We collected the words with the help of synonym finders.^6^ ^,7^ The sample contained five nouns (including the nouns from Study 1) and nine verbs for each category (see Appendix Table A2). Some of the selected words came from a fighting and military context (e.g., invasion, to rush forward), others related to non-physical actions (e.g., to affront or to dissent), and many could be used in a physical as well as a non-physical sense (e.g., attack; and resistance). All words had been checked for ambiguity and comprehensibility. In addition, and mixed with the defense and attack words the words “agieren” (translated: to act/to operate) and “reagieren” (translated: to react) were presented.

On each page, participants saw one of the words and two arrows, one representing a left-to-right directionality and one arrow representing a right-to-left directionality. The words were presented in random order for each participant. Participants had to drag and drop the words onto the arrow that they thought more appropriate. The arrow on which the word was positioned was our binary dependent variable. Words that were not clearly positioned on one of the arrows (i.e., the space between, below or above) were coded as missing data.

Once participants had assigned all words to the arrows, we collected sociodemographic data and informed participants about the aim of the study.

### Results

As the positioning of the words was nested within participants we conducted a binary logistic regression analysis by means of GEE (Wilson and Lorenz, 2015). The binary coding of the position served as our criterion variable (positioning on the arrow showing from right to left = 0; positioning on the arrow showing from left to right = 1). We included word type (dummy coded: attack word = 0; defense word = 1) as predictor.

**FIGURE 1:**
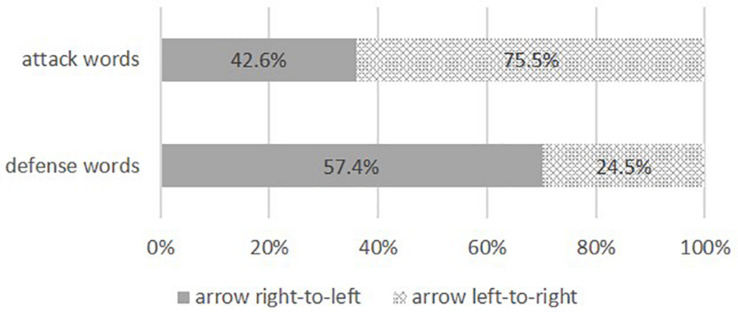
Percentage arrow alignment Study 2.

In line with our hypothesis and Study 1, participants predominantly positioned the attack words on the arrow with a left-to-right trajectory [75.5%, see Figure 1; *b* = 1.13, *SE* = 0.14, Wald’s-χ^2^(1) = 61.08, *p* < 0.001, OR = 3.08, 95% CI (2.33, 4.09)].

Defense words were less likely positioned on the arrow with a left-to-right direction compared to the attack condition [42.6%; *b* = −1.42, *SE* = 0.21, Wald’s-χ^2^(1) = 44.87, *p* < 0.001, OR = 0.24, 95% CI (0.16; 0.37)]. A simple intercept analysis revealed that, in line with the reversal hypothesis, participants preferred to position the defense words on the arrow with a right-to-left trajectory [57.4%; *b* = −0.30, *SE* = 0.10, Wald’s-χ^2^(1) = 8.02, *p* = 0.005, OR = 0.74, 95% CI (0.61; 0.91)].

For the generic words denoting action and reaction (“agieren”, translated: to act/to operate and “reagieren”, translated: to react), we ran an additional GEE (criterion variable: positioning on the arrow showing from right to left = 0; positioning on the arrow showing from left to right = 1; predictor, dummy coded: “agieren” = 0; “reagieren” = 1).

In direct contrast to the opposite directions of words associated with attack and defense, the generic words denoting action and reaction (“agieren”, translated: to act/to operate and “reagieren”, translated: to react) both reflected a classic SAB. Both were clearly positioned on an arrow directed from left-to-right [“agieren”: 77.3%, *b* = 1.23, *SE* = 0.24, Wald’s-χ^2^(1) = 25.59, *p* < 0.001, OR = 3.41, 95% CI (2.125.48); simple slope analysis for “reagieren”: 70.3%, *b* = 0.86, *SE* = 0.22, Wald’s-χ^2^(1) = 15.65, *p* < 0.001, OR = 2.37, 95% CI (1.54; 3.63)].

#### Binomial Tests on Word Level

We took a closer look at the individual words and ran binomial tests for each of the words (see Appendix Table A2). All of the attack words were more likely positioned on the arrow with a left-to-right trajectory. However, the picture is not so clear-cut for the defense words which were expected to be positioned on the arrow with a right-to-left directionality. Although 13 of the 16 words showed the reversal, this was only significant for five words. Of the three words that did not show a reversal one (“kontern”, translated “to retaliate”) actually showed a significant left-to-right bias. Apparently, the spatial representation of defense with a right-to-left trajectory is not as strong and unequivocal as the spatial representation of attack with a left-to-right trajectory is.

### Discussion

On the one hand, the results clearly support the notion that defensive actions tend to show a bias for a right-left direction. Yet, the spatial representation directed to the left was much weaker and less unequivocal than that of attack as directed to the right. Still, these results suggest that the spatial representations of defense and attack are located in different positions with different trajectories. On the other hand, there is no evidence that the reversal of the SAB for the concept of defense is due to its responsive nature. The positioning of the generic verb “to react” falsifies the assumption of actions that represent reactions as being spatially represented as directed from right-to-left. The words denoting action and reaction were equally clearly positioned as left-to-right. Further research might test this assumption of a spatial representation of response actions with a wider set of concepts.

One explanation for the different positioning and trajectories of attack and defense might be the fact that the concepts involve different levels of power and dynamics. Many of the attack words involved movement in some form whereas hardly any of the defense words did (with the possible exception of “kontern”, translated: to retaliate, which actually showed the reverse bias). Thus, it might be these aspects of agency, power and dynamics that drive the classic SAB.

**FIGURE A3:**
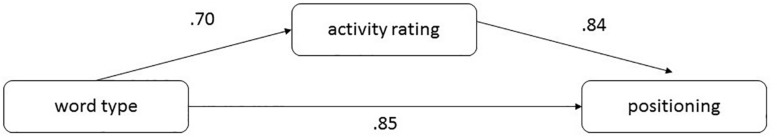
Mediation of activity ratings on the effect of word type on positioning. *Note* Paths of the mediation analysis with the coefficients of the zero-order correlations.

To look into this more deeply we had new participants rate the words presented in Study 2. These words were intermixed with ten new additional synonyms for attack and defense for a larger pool. For each word participants (*N* = 29, 65.51% female, *M*_*age*_ = 28.03, *SD*_*age*_ = 9.59) moved a slider along a continuum (0–100) from passive to active. On average the 16 defense words of Study 2 were rated as less active than the 16 attack words of Study 2 [*M*_*attack*_ = 78.53, *SD*_*attack*_ = 11.63; *M*_*defense*_ = 55.52, *SD*_*defense*_ = 15.26, *t*(28) = 8.78, *p* < 0.001]. The same was true for defense and attack words when we included the new words [*M*_*attack*_ = 76.61, *SD*_*attack*_ = 11.06; *M*_*defense*_ = 52.06, *SD*_*defense*_ = 15.29, *t*(28) = 9.38, *p* < 0.001]. Moreover these ratings also partially mediated the choice of arrows for defense and attack words in Study 2 (indirect effect: *b* = 0.13, *SE* = 0.02, *z* = 8.13, *p* < 0.001, direct effect: *b* = 0.19, *SE* = 0.02, *z* = 8.21, *p* < 0.001, see also Appendix Figure A3).

## General Discussion

Two studies tested how the concepts of attacking and defending are spatially represented. According to the SAB (Suitner and Maass, 2016), we expected that attacking is seen as an agentic behavior and therefore associated with a left-to-right trajectory. Indeed, our data find support for this prediction: In Study 1, participants’ artworks depicting an attack represented the galaxy attacking on the left-hand side of the galaxy defending (and therefore with a left-to-right trajectory) and in Study 2, attack words were preferably aligned on the arrow showing from left-to-right. In contrast, the spatial representation of defense did not show the classic SAB. In Study 1, the defending space ship was placed about 50:50 on the left and right-hand side of the pictures which means that in line with our hypothesis the original SAB effect was considerably reduced and even eliminated. But with a more abstract operationalization in which possible further influences (such as the spatial relevance bias, Halicki et al., 2021) were removed, Study 2 showed a clear reversal. Although the spatial representation directed to the left was much weaker and less unequivocal than that of attack as directed to the right, these results nevertheless suggest that the spatial representations of defense and attack are located in different positions.

We had speculated that because defense is always and necessarily the reaction to a prior attack its spatial representation might be oriented in the opposite position and direction as attack. However, although Study 2 found evidence for a reversal and a right-to-left trajectory for defense actions, the data do not support the assumption that this is due to the responsive nature of defense. Rather, defensive actions are not perceived as agentic as actions of attack. Thus, it might be less the concepts of attack and defense that determine the spatial positioning but the specific actions linked to these concepts. To the extent that these involve a high level of autonomy, power and dynamics they would be located with a rightward oriented bias. Given, however, that there is an ecological confound with many defensive actions low in power and movement, defensive actions (and therefore defense) are overall more likely to be positioned on the right and directed to the left.

### Limitations and Future Research

Although the study design with arrow-alignment in Study 2, is a common SAB approach (see Suitner et al., 2017) directionality might have been more salient than in Study 1. It is possible that the concepts of attack and defense are more strongly associated with moving trajectories than with positioning. However, this idea could be tested in future research.

Another issue that awaits future research is suggested by the results of the mediation analysis. Despite the clear results of lower activity of defense words and a reversed spatial representation the mediation analysis also shows that apparently there must be additional causes for the reversal than merely lower agency.

A further limitation addresses the vertical arrangement of the items that were available for positioning in the picture since vertically might imply an impression of hierarchy or relevance (Giessner and Schubert, 2007). However, this problem might not be addressed easily, since any horizontal arrangement (either controlled and balanced or produced by a randomized cloud pattern) might also affect the positioning bias. Furthermore, our evidence to represent attacking on the horizontal axis could have been fostered by the layout of our paradigm (e.g., horizontal laser beams and a limited height of the background picture). Since attack and defense might also be linked to power and competence and power is associated with the vertical axis (Giessner and Schubert, 2007) it is possible that our results might be overridden by a vertical bias, once the paradigm is opened for vertically oriented pictures. Therefore, future research could test whether the horizontal positioning bias is robust in a context where a vertical alignment is more feasible.

We do not claim that identifying an act as attack or as defense exclusively depends on the trajectory in which it is presented. Of course, the context is also responsible for how an action is encoded and how this effects further evaluations. For example, presenting attackers on the right and defenders on the left did not prevent children from recognizing the situation (Geraci, 2020). Future research could address context effects of the spatial representation of attack and defense and if this also affects identification of attacking and defending behavior.

One could also address boundary conditions of the spatial bias and whether characteristics of participants affect the strength of the bias. For example, spatial intelligence (Hegarty, 2011) could possibly amplify the bias. Further, one could investigate whether measured verbal intelligence (Baddeley et al., 1993) affects the bias (especially relevant for results of Study 2). Further, as gender and status (Carnaghi et al., 2014) are also spatially represented, variations of these aspects of the protagonists in the scenes could possibly influence the bias. For example, it could be tested whether an attack from a person or group with a higher status is stronger associated with a rightward oriented bias than an attacker with lower status.

### Practical Implications

Practical implications of a spatial bias for the concept of attack and defense could also be seen in the field of consumer psychology. It has already been shown that consumers are affected by spatial orientations of products (e.g., effects on price and quality expectations, Valenzuela and Raghubir, 2015 or inferences on heaviness, Deng and Kahn, 2009). A fit between the spatial orientation and attack or defense related products or product claims framed as attack or defense could have a beneficial advertising effect. For example, a virus protection could either be framed as attacking the potential threats or as defending an incoming virus and could be positioned accordingly.

## Conclusion

Our results extend and refine previous research on the SAB. Whereas previous research used aggressive activities (e.g., to kick) these were merely examples of any activity. Our results show that it is not the case that any agent (in the sense of actor) is represented on the left and any activity with a left-to-right trajectory but that position and direction is indeed–as the name suggests–dependent on perceived agency.

## Data Availability Statement

The raw data supporting the conclusions of this article will be made available by the authors, without undue reservation https://osf.io/y7k4u/?view_only=0203de4837b843d7a6eb43f3b8aa58c1.

## Ethics Statement

Ethical review and approval was not required for the study on human participants in accordance with the local legislation and institutional requirements. The patients/participants provided their written informed consent to participate in this study.

## Author Contributions

KH: initial idea, writing and editing the manuscript, data analysis, and preparing studies. MI and MJ: preparing and conducting study 1. MM and MS: preparing and conducting study 2. MW: supervision of design development for studies 1 and 2, and supervision of writing the article. MW: initial idea and supervision. All authors contributed to the article and approved the submitted version.

## Conflict of Interest

The authors declare that the research was conducted in the absence of any commercial or financial relationships that could be construed as a potential conflict of interest.

## Footnotes

1 Although the representation of small numbers and agents on the left and large numbers and recipients of actions on the right may be explained neurologically (Chatterjee et al., 1999; Feigenson et al., 2004; Gevers et al., 2006) these effects have been shown to be culturally flexible and dependent on script direction (e.g., Dehaene et al., 1993; Shaki et al., 2009 for the SNARC effect: Maass and Russo, 2003 for the SAB; see also Román et al., 2015 for a more general model of script direction in the construal of mental representations). Thus, left-to right trajectory for actions that can be observed in cultures reading and writing from left to right reverses in cultures with right-to-left script direction.

2 As our studies were conducted with participants of a culture with left-to-right script direction in the following we refrain from spelling out the mirrored hypotheses for cultures with right-to-left script direction.

3 A sensitivity analysis revealed that the final sample sizes of each study were sufficient to detect a significant effect with pH1 = 0.61, α = 0.05, and 1-β = 0.80.

4 https://www.surveycircle.com/de/

5 https://www.soscisurvey.de/

6 www.duden.de

7 www.openthesaurus.de

## Appendix

**TABLE A1:** Positioning within attack and defense scenes in Study 1.

Attack scenes				Defense scenes			
	
Scene *Translation*	% left	binomial test *t*(d*f* = 99)	*p*	Scene *Translation*	% right	binomial test *t*(d*f* = 99)	*p*
Angriff *attack*	70%	4.34	0.000	Verteidigung *defense*	43%	1.41	0.163
Offensive offense	68%	3.84	0.000	Defensive *defense*	51%	−0.20	0.843
Invasion *invasion*	67%	3.60	0.001	Widerstand *resistance*	51%	−0.20	0.843
Überfall assault	60%	2.03	0.045	Deckung *cover*	49%	0.20	0.843

**Note N* = 100. Binomial test against 50%.*

**TABLE A2:** Alignment of attack and defense words in Study 2.

Attack words				Defense words			
	
Original item	%→	Binomial test *t*(d*f*)	*p*	Original item	%←	Binomial test *t*(df)	*p*
Angriff *attack*	78%	6.84 (100)	0.000	Verteidigung *defense*	58%	1.63 (97)	0.106
Offensive *offense*	88%	11.64 (99)	0.000	Defensive *defense*	76%	6.06 (102)	0.000
Invasion *invasion*	76%	6.27 (101)	0.000	Widerstand *resistance*	54%	0.71 (96)	0.480
Überfall *assault*	71%	4.50 (98)	0.000	Deckung *cover*	74%	5.44 (99)	0.000
Einmarsch *march-in*	77%	6.49 (100)	0.000	Gegenwehr *fightback*	49%	−0.01 (98)	0.921
Attackieren *To attack*	78%	6.73 (99)	0.000	Abwehren to repel/to fend	66%	3.32 (101)	0.001
Anstürmen *To charge*	80%	7.58 (100)	0.000	Blockieren to block	72%	4.64 (94)	0.000
Bekämpfen *To fight against*	78%	6.61 (98)	0.000	Widersetzen to defy	61%	2.34 (100)	0.021
Vorstürmen *To rush forward*	86%	10.28 (103)	0.000	Kontern *to retaliate*	37%	−2.58 (98)	0.011
Umstürzen *To subvert*	61%	2.15 (98)	0.034	Standhalten *To resist/to withstand*	57%	1.37 (90)	0.174
Vorstoßen *To protrude*	82%	8.22 (102)	0.000	Dagegenhalten *to stand/to argue*	47%	−0.61 (95)	0.543
Beschimpfen *To insult*	69%	4.03 (101)	0.000	Widersprechen *To dissent*	54%	0.89 (100)	0.373
Bedrohen *To threaten sb*.	73%	5.08 (101)	0.000	Trotzen *To defy/to beard*	58%	1.53 (96)	0.128
Behaupten *To assert*	75%	5.64 (98)	0.000	Rechtfertigen *To justify*	57%	1.31 (98)	0.193
Vorwerfen *To blame*	75%	5.86 (103)	0.000	Erwidern *To reply/to speak to the contrary*	42%	−1.61 (99)	0.110
Beschuldigen *To accuse*	62%	2.46 (99)	0.016	Beschützen *To protect*	55%	1.02 (95)	0.310

**Note* Binomial test against 50%, diverging degrees of freedoms due to missing data (e.g., word positioned between, above or below arrow).*
